# Pleistocene Landscape Dynamics Drives Lineage Divergence of a Temperate Freshwater Fish *Gobio rivuloides* in Coastal Drainages of Northern China

**DOI:** 10.3390/genes14122146

**Published:** 2023-11-27

**Authors:** Xiaomin Ni, Yun Chen, Guangmin Deng, Cuizhang Fu

**Affiliations:** Ministry of Education Key Laboratory for Biodiversity Science and Ecological Engineering, Coastal Ecosystems Research Station of the Yangtze River Estuary, Institute of Biodiversity Science and Institute of Eco-Chongming, School of Life Sciences, Fudan University, Shanghai 200438, China; 18110700109@fudan.edu.cn (X.N.); 20110700132@fudan.edu.cn (Y.C.); 21110700097@m.fudan.edu.cn (G.D.)

**Keywords:** Gobioninae, time tree, fossil calibration, population demography, river network, conservation unit

## Abstract

Understanding historical processes underlying lineage distribution patterns is a primary goal of phylogeography. We selected *Gobio rivuloides* (Cypriniformes: Gobionidae) as a model to improve our knowledge about how intraspecific genetic divergence of freshwater fishes arises in coastal drainages of northern China via statistical analysis using cytochrome *b* gene. The time-calibrated phylogeny of *G. rivuloides* showed the divergence of two major lineages (I and II) at ~0.98 Ma (million years ago). Lineage I can be divided into two sub-lineages (I-A and I-B) with a divergence time of ~0.83 Ma. Sub-lineage I-A inhabits the Amur River, and sub-lineage I-B lives in the Luan River and Liao River. Lineage II is distributed in the Yellow River and Hai River, with close genetic relationships between the two drainages, and can be split into two sub-lineages (II-C and II-D) with a divergence time of ~0.60 Ma. Our findings indicate that the splitting of lineages and sub-lineages could be attributed to geographic isolation caused by the formation of the Bohai Sea, river capture, and the episodic hydrologic closing of a paleolake during the late Lower–Middle Pleistocene. It is also the first report we know of displaying a clear phylogeographic break for freshwater fishes across coastal drainages in northern China.

## 1. Introduction

Understanding the historical processes that underlie the patterns of lineage distributions is a primary goal of the study of phylogeography [1,2]. Obligate freshwater fishes are restricted to river basins, and their dispersal and colonization across isolated drainages rely on river connectivity [3]. River networks are dendritic habitats that change in time and space, profoundly affecting geographical distributions and the genetic divergence of freshwater fishes [4,5]. Growing evidence suggests that changes to the drainage landscape due to tectonic activity, climatic variation, and erosion through different rock types are the major driving forces of lineage divergence in freshwater fishes (e.g., [6,7,8,9,10,11,12]). Two common types of landscape changes in drainages are river capture and paleo-drainage connection. River capture is a geomorphological process in which portions of catchments are displaced between adjacent drainages because of tectonic uplift or headwater erosion [13]. Paleo-drainage connection is another geomorphological process in which coastal drainages are connected by river coalescence during the Pleistocene period of lowered sea levels [14,15]. River capture and paleo-drainage connection have been used in numerous phylogeographic studies to explain phylogeographic breaks or the lack of phylogenetic structure in freshwater fishes across currently isolated coastal drainages (e.g., [16,17,18,19,20,21,22,23]).

**Figure 1:**
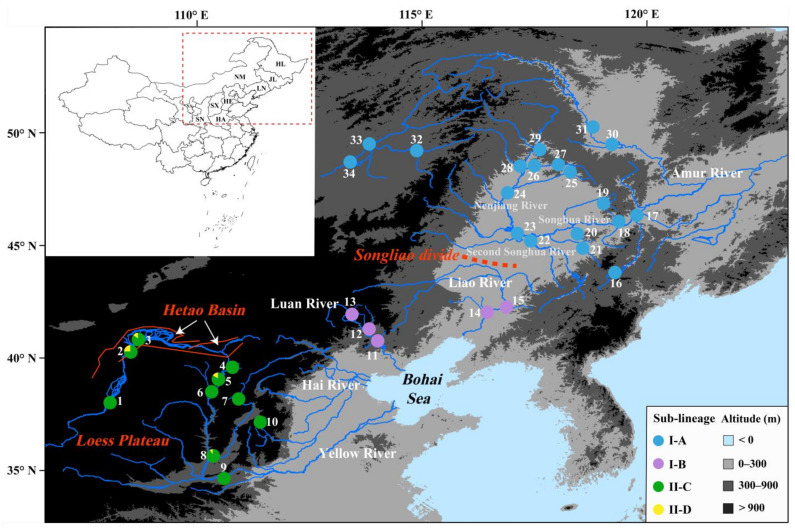
Map showing the distribution of 34 sampling localities and 4 sub-lineages for *Gobio rivuloides*. The detail of each locality is shown in Table S1. Colors correspond to the division of sub-lineages. SN—Shaanxi Province; HA—Henan Province; NX—Ningxia Hui Autonomous Region; SX—Shanxi Province; HE—Hebei Province; NM—Inner Mongolian Autonomous Region; LN—Liaoning Province; JL—Jilin Province; HL—Heilongjiang Province. The map was sourced from National Catalogue Service for Geographic Information (https://mulu.tianditu.gov.cn/ accessed on 1 September 2023).

The Bohai Sea (Figure 1) is the youngest shallow marginal sea of the Asian continent, with an average depth of about 18 m [24]. The timing of the formation of the Bohai Sea is ~0.8–0.89 million years ago (Ma), based on geological evidence [25,26,27]. The Yellow, Hai, Luan, and Liao rivers flow into the Bohai Sea (Figure 1). Before the formation of the Bohai Sea, the paleo-Yellow, Hai, Luan, and Liao rivers could be connected by a long-standing vast freshwater lake—the “Bohai Paleolake”—in this region [25]. After the formation of the Bohai Sea, a substantial decline in sea level could expose the Bohai Sea floor during glacial periods of the Middle–Upper Pleistocene [28], providing connectivity to the currently isolated coastal drainages throughout this region. The findings of a few phylogeographic studies suggest that the Pleistocene glacial cycles giving rise to episodic paleo-drainage connections resulted in the lack of phylogeographic structure in freshwater fishes inhabiting the river basins surrounding the Bohai Sea [29,30]. However, no study has so far demonstrated the influence of the Bohai Sea formation on the lineage divergence in freshwater fishes of northern China.

The Amur River runs ~4370 km before draining into the Tatar Strait between the Okhotsk Sea and the Japan Sea [31]. Geological studies of the past drainage evolution indicated that the Liao River and the three tributaries of the Amur River (Nenjiang River, Songhua River, and Second Songhua River (Figure 1)) had belonged to the same hydrological system flowing southward into the region of the modern Bohai Sea during the Lower Pleistocene (before ~0.94 Ma; [32,33]). The paleo-Songhua, Second Songhua, and Nenjiang rivers were reorganized into the Amur River via river capture between ~0.94 and 0.46 Ma, caused by headward erosion and tectonic uplifting of the Songliao divide [33]. Several studies have shown that the vicariant events via river capture between the Liao River and the Amur River could result in lineage divergence and secondary contact of freshwater fishes across the two drainages [18,34,35].

The Yellow River is the second longest river in China, flowing a length of ~5464 km before emptying into the Bohai Sea. The formation of the modern Yellow River occurred ~1.25–1.5 Ma based on river terrace evidence [36,37]. The upper reaches of the Yellow River originate in the northeastern Tibetan Plateau. It flows over the Chinese Loess Plateau, passing through the Hetao Basin, which forms the middle course of the Yellow River [37,38,39]. The Hetao Basin, with an average altitude of ~900–1200 m, borders the end reaches of the upper Yellow River, where a vast open “Hetao paleo-lake” existed during the Lower–Upper Pleistocene [38,40]. Geological evidence indicates that the uplift of the Hetao Basin outlet occurred ~0.8–0.47 Ma, resulting in the intermittent development of a closed “Hetao paleo-lake” during this period [39,41,42]. Therefore, we hypothesize that the flow of the Yellow River through the Hetao Basin could have been interrupted by the episodic occurrence of a hydrologically closed “Hetao paleo-lake” during the late Lower–Middle Pleistocene, thus facilitating lineage divergence in fish populations of the upper and middle–lower reaches of the Yellow River.

*Gobio rivuloides* (Nichols, 1925) (Cypriniformes: Gobionidae) is a small-sized (<~14 cm in body length) temperate freshwater fish endemic to the coastal drainages of northern East Asia, mainly distributed in the Yellow, Hai, Luan, and Liao rivers surrounding the Bohai Sea, and the Amur River flowing into the Tatar Strait between the Okhotsk Sea and the Japan Sea [43]. It prefers to inhabit the middle and bottom layers of running water of streams and rivers and feeds on benthic invertebrates [44]. *G. rivuloides* reach sexual maturity at two years old, breeding from May to June [44].

Despite numerous phylogeographic studies of the influence of past drainage connections on the patterns of lineage distributions in southern China (e.g., [23,45,46,47,48,49]), our understanding of how changes in the drainage landscape have shaped the phylogeographic patterns of freshwater fishes in northern China is still greatly limited. Using *G. rivuloides* as a model and the statistical analysis of phylogeography based on the cytochrome *b* gene, the present study attempts to further our understanding of the origins of the intraspecific genetic divergence of freshwater fishes in northern China. The specific hypotheses regarding major forces driving the lineage divergence of *G. rivuloides* are tested by examining the formation of the Bohai Sea, river capture between the Liao River and Amur River, and episodic existence of a hydrologically closed “Hetao paleo-lake” in the Yellow River basin.

## 2. Materials and Methods

### 2.1. Specimen Sources

We used 286 individuals obtained from 34 localities of 5 river basins (the Yellow, Hai, Luan, Liao, and Amur rivers) covering the distribution area of *G. rivuloides* (Figure 1; Table S1). All specimens were collected from July 2011 to December 2019 using small set nets and gill nets with the help of local fishermen. Specimens were anesthetized by immersion in an aqueous solution of eugenol (0.25 mL/L), following the laboratory animal guidelines for the ethical review of animal welfare in China (GB/T 35892-2018 [50]), and then stored in 95% ethanol at the Zoological Museum of Fudan University.

### 2.2. Acquisition, Processing, and Statistical Analysis of Sequence Data

A small amount of muscle tissue was cut from each fish specimen and was used for genomic DNA extraction under high salt extraction procedures [51]. Primer pairs 1–13 (Table S2) were used to amplify the complete mitochondrial genome of *G. rivuloides*. The amplification reactions (50 μL) with 25.0 μL 2 × Es Taq MasterMix, 2.0 μL forward primers (20 μM), 2.0 μL reverse primers (20 μM), 2.0 μL genomic DNA, and 19.0 μL dd H_2_O were run under the following program: an initial denaturing at 94 °C for 5 min, 35 cycles of 94 °C for 50 s, 51.8–56.0 °C for 60 s (annealing temperature for each primer pair listed in Table S2), 60 °C for 70s, and a final extension at 70 °C for 10 min. The purification and Sanger sequencing of amplification products was entrusted to Jieli Biology Co., Ltd., Shanghai, China.

The sequences were assembled using Sequencher v5.4 (Gene Codes Corp., Ann Arbor, MI, USA) and then aligned with Mafft v7.427 [52]. The aligned sequences were checked to ensure no indels or stop codons using DAMBE v6.4.100 [53], and their basic characteristics were investigated with MEGA v7.0.26 [54]. *Cyt b* haplotypes for *G. rivuloides* were generated using DnaSP v6.12.01 [55] and were subsequently used in the analyses.

### 2.3. Phylogeny and Ancestral Area Reconstruction

In the absence of a known genus *Gobio* fossil, we adopted the methods of previous studies [21,49] by reconstructing the time-calibrated Bayesian phylogeny of *G. rivuloides* and its close relatives to gain a secondary calibration point in BEAST v2.6.7 [56]. The Leuciscide were selected as the outgroup taxa of the Gobionidae based on the results of previous studies using multi-loci nuclear data [57,58] or mitochondrial genome data [59]. A dataset of 13 protein-coding genes from 38 mitochondrial genomes containing 34 gobionid species and 4 leuciscid species (detailed in Table S3) was used as input. Two fossil records, the earliest known gobionid and *Gnathopogon* fossil (detailed in Table S4), were applied to calibrate the split of families Gobionidae and Leuciscidae and the split of genera *Gnathopogon* and *Coreoleuciscus*, respectively. Each gene was treated as a partition, and its optimal substitution model was obtained through an automatic search using bModelTest [60]. The relaxed log-normal clock model was chosen as the molecular clock, and the birth–death process was set prior as a tree. Two runs of fifty million MCMC generations with a sampling frequency of one thousand were performed. The resulting tree set was combined with the first 30% as a burn-in. ESS > 200 for each parameter was used as a criterion to determine the convergence in Tracer v1.7.0 [61]. The divergence time between *G. rivuloides* and *Gobio coriparoides* (2.69 Ma with a 95% confidence interval of 3.11–2.27 Ma, Figure S1) was obtained as a secondary calibration point, so we constructed the time-calibrated phylogeny of *G. rivuloides* using a normal distribution setting with *m* = 2.69 and *s* = 0.21. The hypothesis tests of the molecular clock using the likelihood ratio test in DAMBE v7.0.35 [62] did not reject the null hypothesis (*X*^2^ = 80.30, *df* = 84, *p* = 0.59), so the strict clock model was selected. *G. coriparoides* was the outgroup. The remaining settings were as described above.

An evolutionary network for haplotypes was generated using the median-joining network method in Network v10.2 [63] and then embellished with the drainage distribution of each haplotype.

Ancestral area estimation was performed under DEC (dispersal–extinction–cladogenesis), DIVALIKE (dispersal–vicariance-like), BAYAREALIKE (Bayesian historical area reconstruction-like; [64]), and their derived model with a *j* (jump dispersal) parameter using the R package BioGeoBEARS v0.2.1 [65]. The input tree was the time-calibrated phylogeny of *G. rivuloides* obtained in this study. Each of the five drainages was regarded as a biogeographical unit and was assigned to each haplotype according to sampling information (Table S1). The Akaike information criterion (AIC) was applied to evaluate the best-fitting biogeographical model.

### 2.4. Genetic Structure and Demographic History

Arlequin v3.5.2.2 was applied to estimate four genetic diversity parameters, i.e., haplotype number, private haplotype number, nucleotide diversity, and haplotype diversity [66]. Analysis of molecular variance and pairwise Φ_ST_ comparison between drainages were also performed in Arlequin. The spatial analysis of molecular variance (SAMOVA) was analyzed to identify the optimal grouping with maximum Φ_ST_ using SAMOVA v2.0 [67].

To detect the expansion signal, neutrality tests were performed to estimate Tajima’s *D* [68] and Fu’s *Fs* [69], and mismatch distribution analyses were conducted with the population expansion model in Arlequin v3.5.2.2. Bayesian skyline plots (BSP) were constructed with *Cyt b* mutation rate of 0.70% substitutions per site per million years (obtained from the time-calibrated phylogeny of *G. rivuloides*) to infer historical demography, using BEAST v2.6.7 and Tracer v1.7.0 [61]. Based on the generation time of two years for *G. rivuloides* [44] and the mutation rate unit of per site per million years used in this analysis, the original values measured on the y-axis on BSP were transformed into effective population size by multiplying them by one million and dividing by two.

**Figure 2:**
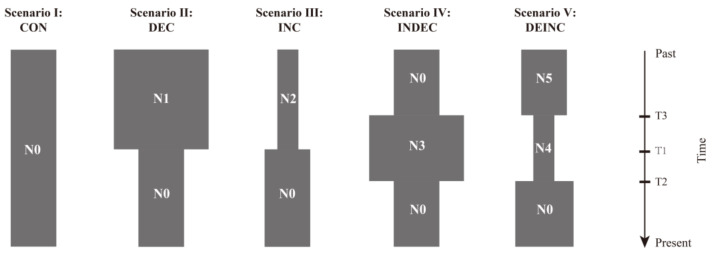
Schematic representation of demographic scenarios tested using DIYABC. Effective population size (N0–N5) and time (T1–T3) are not to scale.

To explore the demographic history of *G. rivuloides*, approximate Bayesian computation (ABC) analyses were performed using DIYABC v.2.1.0 [70]. Five demographic scenarios (scenario I–V) were defined (Figure 2; [71]): a constant population size (scenario I: CON model), a recent bottleneck event (scenario II: DEC model), a recent population expansion (scenario III: INC model), a population expansion followed by a bottleneck event (scenario IV: INDEC), and a bottleneck event followed by a population expansion (scenario V: DEINC). Information on parameters and priors for all scenarios is provided in Table S5. The number of simulated data sets for demographic scenarios was set as 500,000. All summary statistics available in DIYABC were calculated for observed and simulated data sets. The posterior probability (PP) with 95% confidence interval (CI) was computed for each scenario using the logistic regression approach.

## 3. Results

### 3.1. Phylogeny and Ancestral Area Reconstruction

**Figure 3:**
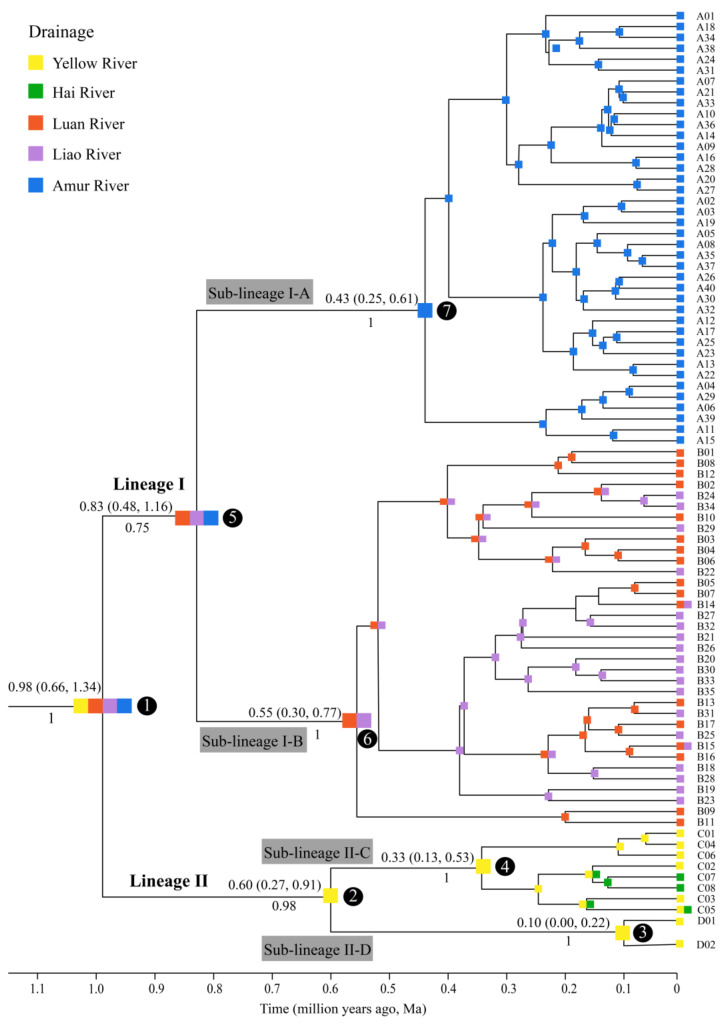
Time-calibrated Bayesian phylogeny and ancestral area reconstruction of 85 *Cyt b* haplotypes for *G. rivuloides*. Ancestral areas were inferred under the DEC+J model. Divergence time (average value and 95% confidence interval) and posterior probability are given above and below the branch, respectively. Codes for the primary nodes are given in a black circle.

A total of 286 *Cyt b* sequences (1140 bp) obtained from 34 sampling localities across the 5 river basins (Figure 1; Table S1) defined 85 haplotypes (GenBank number: OP354001–OP354075, OP354077–OP354086). These sequences contained 62 parsimony informative sites and 44.8% GC content. The time-calibrated phylogeny showed that all populations of *G. rivuloides* could be divided into two major lineages, I and II, and their divergence time occurred ~0.98 Ma with 95% CI (confidence interval) of 1.34–0.66 Ma (Figure 3). Lineage I could be further split into two sub-lineages, I-A and I-B, with a divergence time of ~0.83 Ma (95% CI: 1.16–0.48 Ma). Lineage II comprised sub-lineages II-C and II-D, with a divergence time of ~0.60 Ma (95% CI: 0.91–0.27 Ma). 

**Figure 4:**
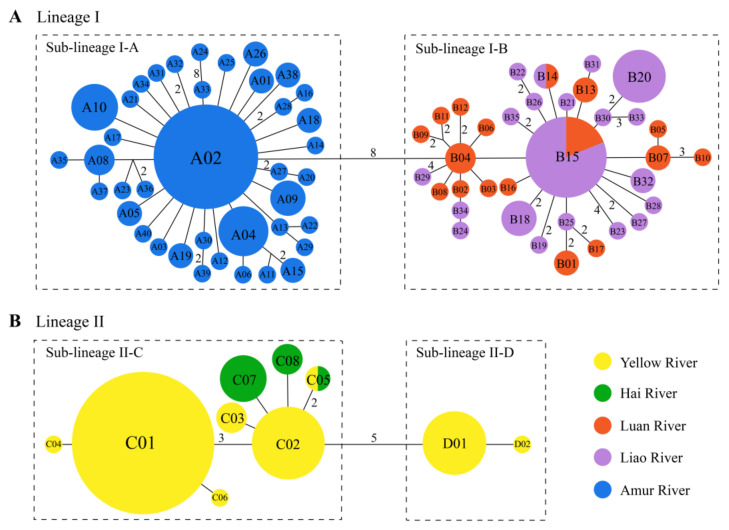
Median-joining networks of *G. rivuloides*: (**A**) lineage I and (**B**) lineage II. The area of each circle is proportional to the sample size for each *Cyt b* haplotype. Values between haplotypes represent mutation steps, and the value of one mutation step is omitted.

The haplotype networks of lineages I and II are displayed in Figure 4. The two major lineages were linked with four mutation steps (not shown in Figure 4). In lineage I, sub-lineages I-A (40 haplotypes) and I-B (35 haplotypes) were connected with eight mutation steps (Figure 4A). The former was distributed in the Amur River. The latter lived in the Luan and Liao rivers with two shared haplotypes, B14 and B15. In lineage II, sub-lineages II-C (eight haplotypes) and II-D (two haplotypes) were linked with five mutation steps (Figure 4B). Sub-lineage II-C occurred in the Yellow and Hai rivers with one shared haplotype, C05, whereas sub-lineage II-D only appeared in the Yellow River.

Ancestral area reconstruction of *G. rivuloides* used the best-fitting DEC+J model as the biogeographical model (Table S6). *G. rivuloides* originated in the Yellow, Luan, Liao, and Amur rivers. Two major vicariant events occurred at nodes 1 and 5, as well as multiple dispersal events within nodes 4 and 6 (Figure 3).

### 3.2. Genetic Structure of Drainage Populations

The overall haplotype diversity (*h*) of *G. rivuloides* was 0.9204, and nucleotide diversity (*π*) was 0.0112 (Table 1). Among the five drainages, the haplotype diversity was in the range of 0.5541 (Yellow River) to 0.9600 (Luan River), and nucleotide diversity was in the range of 0.0011 (Hai River) to 0.0025 (Luan River and Liao River). 

Total Φ_ST_ was 0.7783 (*p* < 0.001). Pairwise divergences among the five drainages were high, except for the Luan and Liao rivers or the Yellow and Hai rivers (Table 2). The SAMOVA analyses supported three groups, the Yellow + Hai rivers, the Luan + Liao rivers, and the Amur River, as the optimum grouping with the maximum Φ_CT_ of 0.7200.

### 3.3. Historical Demography

The population histories of sub-lineages I-A and I-B were analyzed separately because of the strong phylogeographic structure in lineage I. For lineage II, overall population history analysis was conducted because of the lack of phylogeographic structure (Figure 3 and Figure 4).

The values of Tajima’s *D* and Fu’s *Fs* were both negative and statistically significant for the sub-lineages I-A (*D* = −2.441 (*p* = 0.000); *Fs* = −27.441 (*p* = 0.000)) and I-B (*D* = −2.266 (*p* = 0.000); *Fs* = −26.485 (*p* = 0.000)) and both positive and statistically insignificant for the lineage II (*D* = 0.480 (*p* = 0.694); *Fs* = 0.947 (*p* = 0.705)).

**Figure 5:**
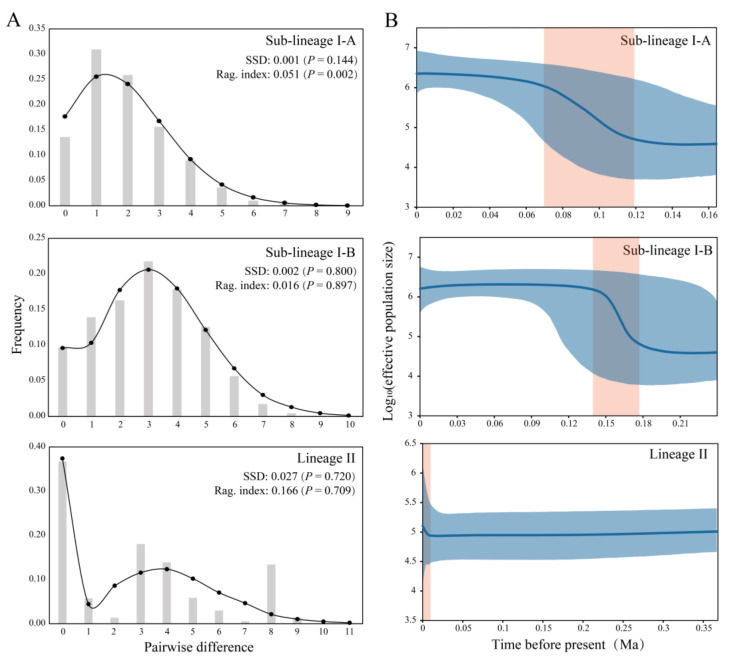
Demographic dynamics for *G. rivuloides*. (**A**) Mismatch distribution and observed and predicted distributions are presented by grey columnar and black lines with dots, respectively. (**B**) Bayesian skyline plot, mid-value, and 95% confidence interval of log_10_ (effective population size) are shown in blue lines and shadows, respectively. Time intervals of demographical expansion are shown in orange shadows.

The mismatch distributions of sub-lineages I-A and I-B exhibited unimodal patterns, and lineage II showed a bimodal pattern (Figure 5A). The BSP analyses indicated that a rapid population expansion occurred in the sub-lineage I-A ~0.118–0.068 Ma, sub-lineage I-B ~0.177–0.140 Ma, and lineage II since 0.012 Ma (Figure 5B).

The ABC analyses (Table S7) demonstrated that sub-lineage I-A favored the DEINC model (scenario V; PP = 0.5679 (95% CI: 0.5567–0.5791)) over the other models, indicating that this sub-lineage underwent a bottleneck event followed by a population expansion. Sub-lineage I-B favored the INC model (scenario III; PP = 0.9805 (95% CI: 0.9787–0.9823)), suggesting it experienced a recent population expansion. Lineage II favored the INDEC model (scenario IV; PP = 0.3480 (95% CI: 0.3425–0.3536)), indicating a population expansion followed by a bottleneck event.

## 4. Discussion

### 4.1. Drivers of Lineage Divergence

In this study, lineages I and II of *G. rivuloides* were revealed to exhibit the pattern of allopatric distributions (Figure 1), with the former’s origin in the Luan, Liao, and Amur rivers and the latter’s origin in the Yellow River, based on the results of our ancestral area reconstruction (Figure 3). Our timing of the splitting between the two major lineages ~0.98 Ma (95% CI: 1.34–0.66 Ma) coincided well with the timing of the Bohai Sea formation during the late Lower Pleistocene [26,27]. Therefore, the divergence between lineages I and II of *G. rivuloides* could be attributed to a vicariant event between the Yellow River and other rivers caused by the formation of the Bohai Sea. Similarly, the splitting of *Rhodeus notatus* populations between China and the Korean Peninsula dated back to the late Lower Pleistocene in a previous study [72] could also result from geographic isolation owing to the formation of the Bohai Sea.

In lineage I, our analyses detected the phylogeographic break of *G. rivuloides* (Figure 1) between the Amur River (sub-lineage I-A) and the other two rivers (Luan and Liao rivers; sub-lineage I-B). Our timing of the divergence between the sub-lineages I-A and I-B (~0.83 Ma) was consistent with the timing of river capture between the upper Amur River and Liao River during the late Lower–Middle Pleistocene [33]. The Amur River currently drains into the Tatar Strait between the Okhotsk Sea and Japan Sea, and the Liao River flows into the Bohai Sea. It suggested that a paleo-drainage connection could not have occurred between the Amur River and Liao River by river coalescence during the Pleistocene period of a substantial decline in sea level. Therefore, the splitting of sub-lineages I-A and I-B of *G. rivuloides* could be attributed to a vicariant event between the Amur River and Liao River via river capture. 

In lineage II, our analyses revealed that sub-lineage II-C originated in the Yellow River and subsequently moved into the Hai River (node 4 in Figure 3). Sub-lineage II-D was restricted to the Yellow River, mainly to the Hetao Basin and its adjacent positions (localities 2, 3, and 5 in Figure 1). Our timing of the divergence between sub-lineages II-C and II-D (~0.60 Ma) aligns with the timing of the episodic closure of “Hetao paleo-lake” during the late Lower–Middle Pleistocene [39,41,42]. Geological evidence suggests that the “Hetao paleo-lake” is not an inland lake but a river-connected lake of the Yellow River [38,39]. Therefore, the splitting of sub-lineages II-C and II-D of *G. rivuloides* could likely be a result of a vicariant event between the upper and middle–lower reaches of the Yellow River caused by the episodic hydrologically closed “Hetao paleo-lake” in the Hetao Basin. A previous study argued that the existence of many isolated paleolakes in the early Lower Pleistocene, before the formation of the modern Yellow River, played an important role in shaping the current fish distribution in the Yellow River basin [73]. 

However, previous phylogeographic studies revealed the lack of phylogeographic structure or secondary contact between divergent lineages of freshwater fishes living in the river basins surrounding the Bohai Sea or across the Liao River and Amur River [18,29,34,35,74,75]. These findings are inconsistent, with a clear phylogeographic break appearing in *G. rivuloides*. Different phylogeographic patterns of freshwater fishes in northern China are likely due to different life histories or dispersal abilities. More research is still needed to build a clear detailed picture of how past landscape dynamics and species traits drive lineage divergence of freshwater fishes in coastal drainages of northern China.

### 4.2. Genetic Structure and Historical Demography

The genetic structure of *G. rivuloides* showed spatial subdivisions in the groups of the Yellow + Hai rivers, the Luan + Liao rivers, and the Amur River. The high degree of genetic differentiation among these three groups (Φ_ST_: 0.7459–0.8358) suggests long-term geographical barriers that prevented gene flow among the populations of different regions [1]. There was a small Φ_ST_ value between the populations of Luan River and Liao River, but only two haplotypes were shared by the two drainages. A similar pattern was also found in the Yellow River and Hai River, with only one haplotype shared by the two drainages. These results indicate the historically close relationships between the populations of the Luan + Liao rivers, as well as between the populations of the Yellow + Hai rivers, suggested by a low level of contemporary gene flow [1]. *G. rivuloides* populations of these groups coalesced ~0.55 Ma and ~0.33 Ma (Figure 3). Geological evidence has shown that the global sea level decreased by ca. 65 m ~0.55 Ma and by ca. 70 m ~0.33 Ma [76]. During the Middle Pleistocene, the Bohai Sea transgression occurred ~0.20 Ma [28]. Therefore, sea level drops during the Middle Pleistocene could have caused a historical confluence of adjacent drainages, including the Luan and Liao rivers as well as Yellow and Hai rivers, resulting in close genetic relationships between the populations of *G. rivuloides* within the group of the Yellow + Hai rivers or the Luan + Liao rivers. The historical exchange of freshwater fish populations among coastal drainages surrounding the Bohai Sea as a result of paleo-drainage connection during the Upper Pleistocene sea level drop has also been indicated by previous phylogeographic studies [29,34,74,75].

The genetic diversity of *G. rivuloides* displayed two patterns (Table 1). One was a pattern with high haplotype diversity (*h*) and low nucleotide diversity (*π*) in the Luan, Liao, and Amur rivers, suggesting these populations experienced recent rapid expansion or bottlenecks followed by population expansion [77]. The other pattern of relatively low *h* and low *π* was detected in the Yellow and Hai rivers and could be attributed to populations experiencing recent bottlenecks [77]. Our ABC analyses further supported these interpretations that sub-lineage I-B underwent population expansion and sub-lineage I-A and lineage II experienced population decline before and after population expansion, respectively.

Our BSP analyses indicated that sub-lineages I-A and I-B underwent rapid population expansion ~0.118–0.068 Ma and ~0.177–0.140 Ma, respectively. Lineage II began to expand around 0.012 Ma. Our timing of *G. rivuloides* rapid population expansion in these three groups coincides well with the periods of warm humid climate in East Asia ~0.18–0.17 Ma, ~0.13–0.1 Ma, and ~0.015 Ma to the present due to intensified East Asian summer monsoons [78,79]. Therefore, *G. rivuloides* may have undergone population decline caused by the cold and dry climate during the glaciation of the Upper Pleistocene, but the subsequent warm and humid climate stimulated the rapid population growth during the Upper Pleistocene interglaciation. 

Two temporal patterns of rapid population expansion prior to the last glacial maximum (LGM, 0.026–0.019 Ma) and after the LGM detected in different lineages of *G. rivuloides* have also been reported in other East Asia freshwater fishes [10,80,81]. However, previous phylogeographic research of freshwater fishes in East Asia offers the strongest evidence of the pattern of rapid population expansion prior to LGM (e.g., [18,21,23,46,49]).

### 4.3. Implications for Conservation

The phylogeographic research evaluates the evolutionary history of species and the genetic structure among populations, providing a valuable reference for the identification of intraspecific conservation units and thus promoting scientific protection and management of unique genetic diversity [82,83,84,85]. Although the overall nucleotide diversity of *G. rivuloides* was high, the nucleotide diversity for each drainage was low (Table 1), pointing to the need to protect the intraspecific genetic diversity of this species. The results of our SAMOVA analysis indicated that three geographical units (Yellow + Hai rivers, Luan + Liao rivers, and Amur River) should be considered as management units (MUs) [86]. According to Kang et al. [87], northern China freshwater fishes were divided into two biogeographical areas—the Heilongjiang Region (Amur River) and the 3H Plain Region (Luan, Liao, Yellow, Hai, and Huai rivers)—based on the freshwater fish fauna. This study identified two lineages in the 3H Plain Region, suggesting substantial differences in the biogeographical divisions at the intraspecific level. Our two identified MUs (Yellow + Hai rivers, Luan + Liao rivers) are located in the 3H Plain Region and show a lack of phylogeographic structure between the Yellow River and Hai River as well as between the Luan River and Liao River (Table 2). Therefore, our results highlight that the Yellow + Hai rivers, Luan + Liao rivers, and Amur River should be considered as three independent areas for spatial conservation of *G. rivuloides* genetic diversity [88].

## 5. Conclusions

*G. rivuloides* exhibited a clear phylogeographic break across coastal drainages in northern China. The splitting of lineages and sub-lineages could be attributed to geographic isolation due to the formation of the Bohai Sea, river capture, and episodic hydrologic closed paleolake during the late Lower–Middle Pleistocene. High haplotype diversity (*h*) and low nucleotide diversity (*π*) in the Luan River, Liao River, and Amur River were due to populations undergoing either recent rapid expansion or bottlenecks followed by population expansion. Relatively low *h* and low *π* in the Yellow and Hai rivers could be attributed to populations experiencing recent bottlenecks. Historical demographic dynamics were likely due to climate oscillations during glacial–interglacial cycles of the Upper Pleistocene. Three genetically distinct geographical units (Yellow + Hai rivers, Luan + Liao rivers, and Amur River) of *G. rivuloides* should be considered separately for conservation and management.

## Figures and Tables

**Table 1:** Genetic diversity for mitochondrial *Cyt b* of *G. rivuloides*.

Drainage	No. of Individuals	No. of Haplotypes	No. of Private Haplotypes	Haplotype Diversity	Nucleotide Diversity
Yellow River	102	8	7	0.5541 ± 0.0495	0.0024 ± 0.0015
Hai River	11	3	2	0.5636 ± 0.1340	0.0011 ± 0.0009
Luan River	25	17	15	0.9600 ± 0.0233	0.0025 ± 0.0015
Liao River	49	20	18	0.8469 ± 0.0412	0.0025 ± 0.0015
Amur River	99	40	40	0.8667 ± 0.0311	0.0018 ± 0.0011
Total	286	85	82	0.9204 ± 0.0103	0.0074 ± 0.0038

**Table 2:** Φ_ST_ (below diagonal) and Bonferroni-corrected *p* values (above diagonal) under pairwise comparisons among the five drainages for *G. rivuloides*.

	Yellow River	Hai River	Luan River	Liao River	Amur River
Yellow River		0.0000	0.0000	0.0000	0.0000
Hai River	0.4017		0.0000	0.0000	0.0000
Luan River	0.7459	0.7658		0.0005	0.0000
Liao River	0.7539	0.7588	0.0834		0.0000
Amur River	0.8145	0.8358	0.7991	0.7987	

## Data Availability

The newly generated sequences in this study are available on GenBank (http://www.ncbi.nlm.nih.gov accessed on 10 April 2023), under accession number OP354001–OP354075, OP354077–OP354086 (*Cyt b* sequences) and OP354223 (mitochondrial genome sequence).

## Supplementary Materials

The following supporting information can be downloaded at: https://www.mdpi.com/article/10.3390/genes14122146/s1, Figure S1: The time-calibrated Bayesian phylogeny of *Gobio rivuloides* and its close relatives inferred from 13 mitochondrial protein-coding genes; Table S1: Geography and *Cyt b* haplotype information of 34 sampling localities for *G. rivuloides*; Table S2: Primer pairs used to amplify the mitochondrial genome of *G. rivuloides*; Table S3: The detailed sources of mitochondrial genomes used in this study; Table S4: Sources, stratigraphic information, and parameter setting of two fossil species used in this study; Table S5: Information of parameters and priors for scenarios tested using DIYABC; Table S6: Model comparison for ancestral area reconstruction of *G. rivuloides* implemented in BioGeoBEARS; Table S7: Posterior probability with 95% confidence interval in bracket for each scenario in DIYABC analyses. References [21,49,89,90,91,92,93,94,95,96,97,98,99,100,101,102,103,104,105,106,107,108,109,110,111,112,113,114,115,116,117,118,119] are cited in the supplementary materials.
