# Supplementary material for: Pleistocene Landscape Dynamics Drives Lineage Divergence of a Temperate Freshwater Fish Gobio rivuloides in Coastal Drainages of Northern China
Source: Genes (Basel). 2023 Nov 27;14(12):2146. doi: 10.3390/genes14122146 (PMC10743038; doi:10.3390/genes14122146)
Supplement: Supplementary file 1 [file genes-14-02146-s001.zip › genes-2711945-supplementary.pdf]

SUPPLEMENTARY MATERIALS

Pleistocene Landscape Dynamics Drives Lineage Divergence of a Temperate Freshwater Fish *Gobio rivuloides* in Coastal Drainages of Northern China

Xiaomin Ni, Yun Chen, Guangmin Deng and Cuizhang Fu *

Ministry of Education Key Laboratory for Biodiversity Science and Ecological Engineering, Coastal Ecosystems Research Station of the Yangtze River Estuary, Institute of Biodiversity Science and Institute of Eco-Chongming, School of Life Sciences, Fudan University, Shanghai 200438, China; 18110700109@fudan.edu.cn (X.N.); 20110700132@fudan.edu.cn (Y.C.); 21110700097@m.fudan.edu.cn (G.D.); czfu@fudan.edu.cn (C.F.)

* Correspondence: czfu@fudan.edu.cn

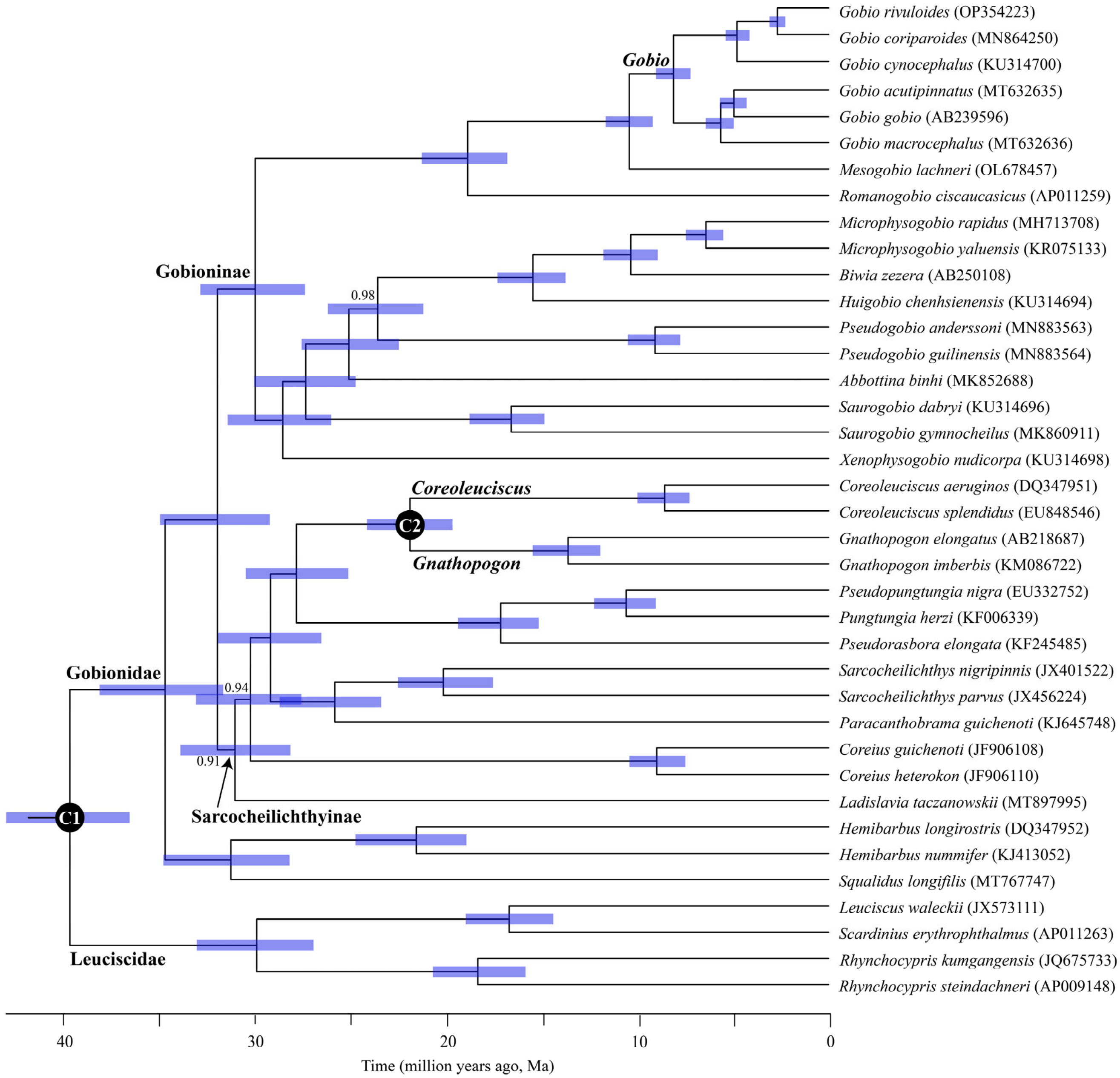

Figure S1. The time-calibrated Bayesian phylogeny of *Gobio rivuloides* and its close relatives inferred from 13 mitochondrial protein-coding genes. C1 and C2 are two fossil calibration points, and their detailed information is given in Table S4. The blue bars show the 95% confidence interval of divergence times. The posterior probabilities are given near the node except for one.

Table S1. Geography and *Cyt b* haplotype information of 34 sampling localities for *G. rivuloides*.

Sampling locality	Coordinate	No.	Cyt *b* Haplotype (No.)
Yellow River			
1. Wuzhong County, Ningxia Hui Autonomous Region	38.03°N, 106.19°E	3	C01(2), C02(1)
2. Dengkou County, Inner Mongolia Autonomous Region	40.32°N, 107.05°E	20	C01(14), C02(1), D01(5)
3. Linhe District, Bayan Nur City, Inner Mongolia Autonomous Region	40.68°N, 107.40°E	20	C01(13), C02(2), C04(2), D02(3)
4. Pianguan County, Shanxi Province	39.58°N, 111.44°E	7	C01(6), C02(1)
5. Baode County, Shanxi Province	39.03°N, 111.08°E	24	C01(14), C02(4), C03(1), C04(1), D01(3), D02(1)
6. Shenmu County, Shaanxi Province	38.53°N, 110.74°E	2	C02(2)
7. Loufan County, Shanxi Province	38.18°N, 111.85°E	3	C01(2), C05(1)
8. Hancheng City, Shaanxi Province	35.61°N, 110.59°E	21	C01(13), C02(5), C06(1), D02(2)
9. Sanmenxia City, Henan Province	34.78°N, 111.19°E	2	C01(1), C02(1)
Hai River			
10. Yushe County, Shanxi Province	37.14°N, 112.94°E	11	C05(1), C07(7), C08(3)
Luan River			
11. Chengde County, Hebei Province	40.77°N, 118.16°E	10	B01(2), B02(1), B03(1), B04(2), B05(1), B06(1), B07(1), B08(1)
12. Longhua County, Hebei Province	41.27°N, 117.67°E	1	B09(1)
13. Weichang County, Hebei Province	41.95°N, 116.94°E	14	B04(1), B07(1), B10(1), B11(1), B12(1), B13(2), B14(1), B15(4) B16(1), B17(1)
Liao River			
14. Xinmin City, Liaoning Province	42.02°N, 122.85°E	25	B14(1), B15(13), B18(1), B19(1), B20(4), B21(1), B22(1), B23(1) B24(1), B25(1)
15.Tieling City, Liaoning Province	42.29°N, 123.84°E	24	B15(4), B18(3), B20(5), B24(1), B26(1), B27(1), B28(1), B29(1) B30(1), B31(1), B32(2), B33(1), B34(1), B35(1)
Amur River			
16. Dunhua City, Jilin Province	43.69°N, 128.60°E	6	A01(1), A02(3), A03(1), A04(1)
17. Yilan County, Heilongjiang Province	46.33°N, 129.55°E	4	A02(1), A05(1), A06(1), A07(1)
18. Tonghe County, Heilongjiang Province	45.98°N, 128.81°E	2	A02(1), A08(1)
19. Tieli City, Heilongjiang Province	46.98°N, 128.04°E	1	A04(1)
20. Harbin City, Heilongjiang Province	45.54°N, 127.01°E	10	A01(1), A02(3), A04(1), A08(1), A09(1), A10(1), A11(1), A12(1)
21. Wuchang City, Heilongjiang Province	44.93°N, 127.09°E	4	A02(1), A13(1), A14(1), A15(1)
22. Songyuan City, Jilin Province	45.17°N, 124.83°E	7	A02(3), A09(1), A16(1), A17(1), A18(1)
23. Daan City, Jilin Province	45.55°N, 124.29°E	1	A04(1)
24. Qiqihar City, Heilongjiang Province	47.34°N, 123.92°E	5	A02(1), A19(1), A20(1), A21(1), A22(1)
25. Beian City, Heilongjiang Province	48.22°N, 126.53°E	6	A02(3), A08(1), A23(1), A24(1)
26. Nehe City, Heilongjiang Province	48.46°N, 124.91°E	4	A02(1), A05(1), A25(1), A26(1)
27. Wudalianchi City, Heilongjiang Province	48.52°N, 126.20°E	6	A02(2), A04(2), A10(1), A15(1)
28. Daur Autonomous Banner of Morin Dawa, Inner Mongolia Autonomous Region	48.48°N, 124.54°E	5	A02(3), A27(1), A28(1)
29. Nenjiang County, Heilongjiang Province	49.24°N, 125.24°E	5	A09(1), A10(2), A19(1), A29(1)
30. Xunke County, Heilongjiang Province	49.60°N, 128.48°E	6	A02(3), A09(1), A30(1), A31(1)
31. Heihe City, Heilongjiang Province	50.22°N, 127.53°E	5	A02(2), A10(1), A32(1), A33(1)
32. Hailar District, Inner Mongolia Autonomous Region	49.27°N, 119.78°E	4	A02(2), A04(1), A10(1)
33. Manzhouli City, Inner Mongolia Autonomous Region	49.38°N, 117.74°E	8	A02(2), A04(1), A18(1), A26(1), A34(1), A35(1), A36(1)
34. Xin Barag Right Banner, Inner Mongolia Autonomous Region	48.65°N, 116.83°E	10	A02(4), A10(1), A37(1), A38(2), A39(1), A40(1)

Table S2. Primer pairs used to amplify the mitochondrial genome of *G. rivuloides.*

Primer pair	Forward primer		Reverse primer		Annealing temperature	Source
	Name	Sequence (5'-3')	Name	Sequence (5'-3')		
1	Gob12SF	AAGGCATGGTCCYGACCTTA	Gob16sR	TTCGGTAGGTCTRTCACTTC	55.4 ºC	[89]
2	GobValF	ACACCGAGAAGACATCCA	GobLeuR	GGGAAGAGGAYTTGAACC	54.3 ºC	[49, 89]
3	GobND1F	GCAGCCGCTATTAAGGGTT	GobND1R1	GGRTTCATTGATGGAGGA	53.3 ºC	[89]
4	SDIleF	GCCCAAGGACCACTTTGATAG	GobCOIR	CCAAATACRAGATARAGGGT	52.5 ºC	[89]
5	AsnF	AGCGAGCATCCATCTACTT	ARSerR	GGTYATGTGACTGGCTTGA	54.6 ºC	[89]
6	CGobCOIF	TGAGAAGCCTTYGCCGCYAAACG	TGobATP6R	GCTTGGTGTGCCATTARACGTTTTCTTG	52.4 ºC	[21, 89]
7	ZXSATP6F	AATCAACCAACMGTAGCYCT	GobArgR	CTGAGYCGAAATCAGAGG	53.3 ºC	[21, 89]
8	GobGlyF	CTTCCAATYATTTAGYCTTGG	GRND4R	CCGGCCCAYCAGATTTTA	51.8 ºC	[49]; this study
9	TGobND4FS	TAGCCAGCCAAAAYCACAT	GobLeuR	TGGAYTTGCACCAAGAGT	54.5 ºC	[21, 89]
10	GobSerF1	ACTYACCRAGGAAGGACA	GobND5R	TCCYCAGGCAAGYCGTTT	54.0 ºC	[89]
11	TGobND5F	GTAGCTTTCTCRACATC	GobCytbR	GGGTGDGTTTTTCGTAG	52.8 ºC	[21]; this study
12	GobND6F	AAAATAGGTCATAATTCTTGCTCGG	GobProR	GTTTAATTTAGAATTCTGGCTTTGG	56.0 ºC	[89]
13	HemDloopF	AAAGCATCGGTCTTGTAATC	HemDloopR	CTTGGCTAGGCGTCTTGG	53.0 ºC	[89]

Table S3. The detailed sources of mitochondrial genomes used in this study.

Genus	Species	Sampling site	Genbank No.	References
Gobio	*Gobio acutipinnatus*	Bu'erjin, Xinjiang Uygur Autonomous Region, China	MT632635	[90]
	Gobio gobio	Plana, Czech Republic	AB239596	[91]
	Gobio macrocephalus	Wangqing County, Jilin Province, China	MT632636	[90]
	Gobio coriparoides	Linxia Hui Autonomous Prefecture, Gansu Province, China	MN864250	[92]
	Gobio rivuloides	Yushe County, Shanxi Province, China	OP354223	this study
	Gobio cynocephalus	Jalaid Banner, Hinggan League, Inner Mongolia, China	KU314700	[93]
Mesogobio	*Mesogobio lachneri*	Linjiang City, Jilin Province, China	OL678457	[94]
Romanogobio	*Romanogobio ciscaucasicus*		AP011259	unpublished
Microphysogobio	*Microphysogobio rapidus*	South Korea	MH713708	[95]
	Microphysogobio yaluensis	South Korea	KR075133	[96]
Biwia	*Biwia zezera*	Shiga, Moriyama, Japan	AB250108	[97]

Huigobio	*Huigobio chenhsienensis*	Ningdu County, Jiangxi Province, China	KU314694	[93]
Pseudogobio	*Pseudogobio anderssoni*	Anyi County, Jiangxi Province, China	MN883563	[98]
	Pseudogobio guilinensis	Pingle County, Guangxi Province, China	MN883564	[98]
Abbottina	*Abbottina binhi*	Pingguo County, Guangxi Province, China	MK852688	[99]
Saurogobio	*Saurogobio dabryi*	Xiushui County, Jiangxi Province, China	KU314696	[93]
	Saurogobio gymnocheilus	Hengdong County, Hunan Province, China	MK860911	[100]
Xenophysogobio	*Xenophysogobio nudicorpa*	Jiangjin District, Chongqing City, China	KU314698	[93]
Coreoleuciscus	*Coreoleuciscus aeruginos**	South Korea	DQ347951	[101]
	Coreoleuciscus splendidus	South Korea	EU848546	[101]
Gnathopogon	*Gnathopogon elongatus*		AB218687	[91]
	Gnathopogon imberbis		KM086722	[102]
Pseudopungtungia	*Pseudopungtungia nigra*	South Korea	EU332752	[103]
Pungtungia	*Pungtungia herzi*	South Korea	KF006339	[104]
Pseudorasbora	*Pseudorasbora elongata*	Wuyuan County, Jiangxi Province, China	KF245485	[105]
Sarcocheilichthys	*Sarcocheilichthys nigripinnis*	Kaihua County, Zhejiang Province, China	JX401522	[106]
	Sarcocheilichthys parvus	Yongjia County, Zhejiang Province, China	JX456224	[107]
Paracanthobrama	*Paracanthobrama guichenoti*		KJ645748	[108]
Coreius	*Coreius guichenoti*	Banan District, Chongqing City, China	JF906108	[109]
	Coreius heterokon	Banan District, Chongqing City, China	JF906110	[109]
Ladislavia	*Ladislavia taczanowskii*	Kuandian County, Liaoning Province, China	MT897995	[110]
Hemibarbus	*Hemibarbus longirostris*	Ganwon-do, South Korea	DQ347952	[111]
	Hemibarbus nummifer	Wuyishan City, Fujian Province, China	KJ413052	[112]
Squalidus	*Squalidus longifilis*	Fengcheng City, Liaoning Province, China	MT767747	[89]
Rhynchocypris	*Rhynchocypris steindachneri*	Yamagata, Japan	AP009148	[113]
	Rhynchocypris kumgangensis	Gangwon Province, Korea	JQ675733	[114]
Leuciscus	*Leuciscus waleckii*	Inner Mongolia, China	JX573111	[115]
Scardinius	*Scardinius erythrophthalmus*		AP011263	unpublished

* The scientific name in Genbank is *Coreoleuciscus splendidus*, and Song and Bang [116] revised it as *Coreoleuciscus aeruginos*.

Table S4. Sources, stratigraphic information, and parameter setting of two fossil species used in this study.

Fossil species	Code	Age of stratigraphy	BEAUti parameter		
			M	S	Offset
† *Palaeogobio zhongyuanensis* [117]	C1	50.5–42.0 Ma [118]	3.82	0.05	0
† *Gnathopogon macrocephala* [117]	C2	20.4–15.0 Ma [119]	2.86	0.08	0

Table S5. Information of parameters and priors for scenarios tested using DIYABC.

Scenarios	Parameter	Distribution	Interval	Constraint
Scenario I (CON model)	N0: the most recent effective population size	Uniform	10^2–$3x10^6$	
Scenario II (DEC model)	N0: the most recent effective population size	Uniform	10^2–$3x10^6$	N1>N0
	N1: the large effective population size	Uniform	10^2–$3x10^6$	
	T1: time of bottleneck effect	Uniform	10^2–$3x10^6$	
Scenario III (INC model)	N0: the most recent effective population size	Uniform	10^2–$3x10^6$	N2<N0
	N2: the small effective population size	Uniform	10^2–$3x10^6$	
	T1: time of population expansion	Uniform	10^2–$3x10^6$	
Scenario IV (INDEC model)	N0: the most recent effective population size	Uniform	10^2–$3x10^6$	N3>N0
	N3: the large effective population size	Uniform	10^2–$3x10^6$	T3>T2
	T2: time of bottleneck effect	Uniform	10^2–$3x10^6$	
	T3: time of population expansion	Uniform	10^2–$3x10^6$	
Scenario V (DEINC model)	N0: the most recent effective population size	Uniform	10^2–$3x10^6$	N4<N0
	N4: the small effective population size	Uniform	10^2–$3x10^6$	N5>N4
	N5: the large effective population size	Uniform	10^2–$3x10^6$	T3>T2
	T2: time of population expansion	Uniform	10^2–$3x10^6$	
	T3: time of bottleneck effect	Uniform	10^2–$3x10^6$	

Table S6. Model comparison for ancestral area reconstruction of *G. rivuloides i*mplemented in BioGeoBEARS. The best-fitting model is in bold.

Model	LnL	Numparams	Dispersal	Extinction	Founder event speciation	AIC	AIC_wt
DEC	-62.64	2	0.13	1.00E-12	0.00E+00	129.30	3.80E-02
**DEC+J**	**-58.66**	**3**	**0.06**	**1.00E-12**	**8.70E-03**	**123.30**	**7.50E-01**
DIVALIKE	-63.54	2	0.19	1.00E-12	0.00E+00	131.10	1.60E-02
DIVALIKE+J	-60.02	3	0.08	1.00E-12	1.10E-02	126.00	1.90E-01
BAYAREALIKE	-84.94	2	0.01	1.70E+00	0.00E+00	173.90	7.90E-12
BAYAREALIKE+J	-66.55	3	0.05	1.00E-07	1.70E-02	139.10	3.00E-04

Table S7. Posterior probability with 95% confidence interval in bracket for each scenario in DIYABC analyses. The highest posterior probability is in bold.

Lineage/Sub-lineage	ScenarioI : CON	Scenario II: DEC	Scenario III: INC	Scenario IV: INDEC	Scenario V: DEINC
Sub-lineage I-A	0.0025 [0.0000,0.0107]	0.0021 [0.0000,0.0104]	0.4250 [0.4137,0.4362]	0.0025 [0.0000,0.0108]	**0.5679 [0.5567,0.5791]**
Sub-lineage I-B	0.0056 [0.0048,0.0063]	0.0044 [0.0038,0.0050]	**0.9805 [0.9787,0.9823]**	0.0055 [0.0048,0.0062]	0.0041 [0.0035,0.0046]
Lineage II	0.2212 [0.2162,0.2262]	0.3333 [0.3277,0.3390]	0.0442 [0.0400,0.0485]	**0.3480 [0.3425,0.3536]**	0.0532 [0.0489,0.0574]
